# Machine Learning-Based Presymptomatic Detection of Rice Sheath Blight Using Spectral Profiles

**DOI:** 10.34133/2020/8954085

**Published:** 2020-10-12

**Authors:** Anna O. Conrad, Wei Li, Da-Young Lee, Guo-Liang Wang, Luis Rodriguez-Saona, Pierluigi Bonello

**Affiliations:** ^1^Department of Plant Pathology, The Ohio State University, Columbus, Ohio, USA; ^2^Department of Food Science and Technology, The Ohio State University, Columbus, Ohio, USA

## Abstract

Early detection of plant diseases, prior to symptom development, can allow for targeted and more proactive disease management. The objective of this study was to evaluate the use of near-infrared (NIR) spectroscopy combined with machine learning for early detection of rice sheath blight (ShB), caused by the fungus *Rhizoctonia solani*. We collected NIR spectra from leaves of ShB-susceptible rice (*Oryza sativa* L.) cultivar, Lemont, growing in a growth chamber one day following inoculation with *R. solani*, and prior to the development of any disease symptoms. Support vector machine (SVM) and random forest, two machine learning algorithms, were used to build and evaluate the accuracy of supervised classification-based disease predictive models. Sparse partial least squares discriminant analysis was used to confirm the results. The most accurate model comparing mock-inoculated and inoculated plants was SVM-based and had an overall testing accuracy of 86.1% (*N* = 72), while when control, mock-inoculated, and inoculated plants were compared the most accurate SVM model had an overall testing accuracy of 73.3% (*N* = 105). These results suggest that machine learning models could be developed into tools to diagnose infected but asymptomatic plants based on spectral profiles at the early stages of disease development. While testing and validation in field trials are still needed, this technique holds promise for application in the field for disease diagnosis and management.

## 1. Introduction

Plant disease diagnosis can be time-consuming and resource-intensive, requiring trained personnel to either scout for disease symptoms in the field or to run laboratory tests ranging from isolation to more modern molecular identification of pathogens [1, 2]. Once diseases are detected, management options may be limited, especially if disease symptoms are widespread, and/or are cost prohibitive. Approaches that require minimal training are relatively inexpensive and have the potential to be used in a rapid and high-throughput manner are attractive alternatives, especially if they are capable of diagnosing diseased plants prior to the development of symptoms [3]. Early detection and diagnosis of plant diseases may allow for targeted disease management, i.e., applying treatments selectively and only to diseased plants rather than applying treatments to an entire area where not all plants may be diseased. This in turn can lead to reductions in the time and money spent managing for plant diseases, since only smaller areas would need to be treated. It may also lead to a reduction in yield losses, if the disease is detected before it has a chance to spread widely. Methods available currently for rapid detection include PCR-based approaches. While there are field-based methods for PCR (e.g., Loop-mediated isothermal amplification) [4], these methods are not always available, require active sampling, and may not be amenable to high-throughput disease diagnosis in the field.

Near-infrared (NIR) spectroscopy is one promising method for rapid and high-throughput classification of diseased plants, providing a potential tool for passive monitoring of plant diseases. In addition, handheld instruments, like the one used in the present study, require minimal training to use and are relatively inexpensive. NIR spectroscopy is a type of vibrational spectroscopy that examines how light interacts with a sample over the 750–2500 nm region of the electromagnetic spectrum [5, 6]. When plants are suffering from an infection, their metabolism is significantly altered. Any wholesale changes in plant phytochemistry can, at least in principle, be detected by way of chemical fingerprints generated with NIR spectroscopy and related approaches, as demonstrated by Fallon et al. [7] for oak wilt, Couture et al. [8] for potato virus Y, and Gold et al. [9] for potato late blight. Transmission and reflectance are two ways in which NIR spectra are collected. With transmission spectroscopy, the detector and infrared source are placed on opposite sides of the sample and radiation passing through the sample is measured, whereas with reflectance spectroscopy the detector and source are on the same side of the sample and radiation that reflects off the sample is measured. For solid samples, such as plant tissues, reflectance spectroscopy is commonly used [5]. Regardless of the method used, each sample has a unique NIR spectrum, a byproduct of its chemical and physical properties [5]. An added benefit of NIR spectroscopy is that it requires minimal sample preparation [6], which can allow for more rapid measurement and subsequent classification (e.g., of diseased plants). Finally, NIR spectroscopy measures chemicals containing the groups -OH, -NH, and -CH [5, 6], which are found in primary and secondary metabolites—key components of plants and plant defenses against pathogens. Variation in NIR reflectance can also be attributed to differences in the water content of samples [10, 11]. Plant moisture content is an indicator of plant health, e.g., by way of desiccation (i.e., wilting) or the formation of water-soaking lesions (due to vacuole collapse), both of which are common symptoms of pathogen infection.

On its own, NIR spectroscopy can be used to understand the chemical and physical properties of a given sample. However, in order to use it for rapid classification [12], it must be combined with some form of predictive modeling, since differences in spectral bands (i.e., wavelengths) may not be obvious. Machine learning (a tool used for artificial intelligence) is one approach for efficiently developing predictive models, particularly when working with large and complex datasets [13], such as the chemical fingerprints collected by spectral methods [14–16]. Support vector machine (SVM), a type of machine learning algorithm, is a supervised classification approach that has been used widely for detection, classification, and prediction of plant diseases [17]. For example, SVM has been used to distinguish between healthy and inoculated sugar beets [18], for disease forecasting of rice blast [19], and to distinguish between different plant diseases in multiple plant pathosystems [18, 20]. Therefore, the objective of this study was to evaluate whether NIR spectroscopy combined with machine learning can be used to classify plants as infected prior to the onset of disease symptoms.

We focused on one of the most economically important diseases of rice (*Oryza sativa* L.), rice sheath blight (ShB), caused by the fungus *Rhizoctonia solani* [21]. Early symptoms of the disease include the formation of ellipsoidal or oblong, water-soaked necrotic lesions along the leaf sheath. Under the right conditions, the fungus can spread upwards quickly, forming lesions on upper leaf parts, and eventually cause plant lodging within seven to ten days that may lead to yield reductions as high as 50% [21, 22]. Since there are no rice cultivars fully resistant to ShB, management options are limited to the use of partially resistant cultivars, although fungicides, cultural practices, and biocontrols are other options [21, 23, 24]. Still, Singh et al. [23] identified the need for “smart farming for early disease detection,” highlighting the use of Unmanned Aerial Systems for early detection of ShB [25]. A complementary approach using NIR spectroscopy may be useful for the detection of ShB before the onset of symptoms (e.g., lesions on the leaf sheath), given that multispectral sensing was capable of detecting ShB in rice under moderate to high levels of disease [26] and was also used to predict ShB severity [25]. In this study, we evaluated the use of NIR spectroscopy combined with machine learning as a tool for early detection of ShB in rice. Our results indicate that this approach can be used to identify infected rice plants as early as one day following inoculation with *R. solani*, and therefore, may be a useful tool for early disease detection in field settings.

## 2. Materials and Methods

### 2.1. Plant Material and Inoculations

Rice cultivar Lemont was grown and inoculated according to the methods of Jia et al. [27] with modifications. In brief, seeds were disinfected with 75% ethanol for one minute and 3% sodium hypochlorite for 30 minutes. After washing with sterilized water five times, seeds were then germinated on ½ Murashige and Skoog medium in petri dishes for eight days at 26°C to obtain uniform seedling growth. Then, six seedlings each were transferred to one 13 cm diameter pot containing Pro-Mix-BX growing medium (Premier Tech Horticulture, Quebec, Canada) and grown in a growth chamber (E15, Conviron, Winnipeg, Canada) for four weeks. The growth chamber was maintained at 26°C with 80% humidity and a 12 hr light/12 hr dark period throughout the duration of the experiment.

A total of 39 pots were prepared and maintained with 13 pots (~78 seedlings) per each of three treatments: control (noninoculated), mock-inoculated with potato dextrose agar (PDA) plugs only, and inoculated with *R. solani* on PDA plugs. Approximately four-week-old seedlings were inoculated or mock-inoculated at the base of the stem with two 0.7 cm diameter plugs of 60–72 hr old *R. solani* isolate B2 mycelium grown on PDA, with the mycelial sides of the plugs placed against the plant stem, or PDA only, respectively. Control plants were left as they were. Following inoculation, all pots were covered with a clean 2 L plastic soft drink bottle with the bottom removed and no cap to maintain the level of humidity necessary for the development of ShB [28]. Pots were maintained in trays filled with water to approximately half the height of the tray. To prevent cross-contamination between samples, pots were separated in different trays based on treatment, with one exception. One pot from each treatment was placed in one tray due to space limitations within the growth chamber. Pots were then placed back into the growth chamber until spectral measurements were collected. An additional experiment was performed comparing only control (noninoculated) and inoculated plants (results reported in the supplementary materials). The same procedure as stated above was followed, with the exception that there were only 12 pots (~72 seedlings) per treatment (control and inoculated), and pots from each treatment were in separate trays in the growth chamber. The experiment with three treatments (control, mock-inoculated, and inoculated) occurred from September–October 2019, while the experiment with two treatments (control and inoculated) occurred from April–May 2019.

### 2.2. Collection of Spectral Data

At one day post-inoculation (dpi), trays containing seedlings were removed from the growth chamber to collect spectral measurements. Soda bottles were removed from each pot just prior to the collection of spectral measurements and were replaced once spectral measurements were completed. For each seedling, a spectrum was collected from the adaxial side of one to two leaves at approximately mid-leaf or the widest part of the leaf, and thus away from the site of inoculation at the base of the stem.

NIR spectra were collected with a NeoSpectra micro handheld spectrometer (SiWare Systems, La Canada, CA, USA) with a two-second collection time and a spectral resolution of 16 nm as measured at 1550 nm. The spectral range of the instrument was 1348–2551 nm. A two-second background measurement was collected every pot (approximately every six seedlings) using a protected gold-coated metallic mirror (Thorlabs Inc., Newton, NJ, USA). The mirror was also used to hold leaves in place against the surface of the sensor during the collection of spectral measurements, with the mirror side facing the sensor in the experiment with three treatments and the backside of the mirror facing the sensor in the experiment with two treatments. Spectra were collected, visualized, and exported using SpectroMOST software (SiWare Systems, La Canada, CA, USA). Trays containing plants were then placed back into the growth chamber once all spectral measurements were completed, and remained in the growth chamber until disease symptoms were measured.

### 2.3. Disease Phenotyping

The presence or absence of disease symptoms, including lesion length, was recorded at five and seven dpi for the experiment with three treatments and at seven and nine dpi for the experiment with two treatments. Dates for the detection of disease symptoms were selected based on the rate of disease development in each experiment, which varied slightly. Due to the humid conditions within the soda bottles, fungal growth was observed on PDA plugs from mock-inoculated plants, although mock-inoculated plants did not develop any stem lesions after seven days.

### 2.4. Data Preprocessing and Analysis

Raw NIR spectra from rice leaves were imported into R version 3.6.0 [29]. Outliers were detected and trimmed based on the method of Heim et al. [30] (dfunc = depth.FM, nb = 10, smo = 0.1, trim = 0.06) (packages: “fda.usc” and “fda”) [31, 32]. In brief, spectra were identified as outliers based on the assumption that the depth of the spectral curve of a sample and the sample's outlyingness are inversely related, such that the depth of a spectral curve of an outlier will be significantly lower [31]. Following outlier detection using a depth-based approach, additional outliers based on boxplots were identified at the wavelength 1772 nm, which was representative of abnormal NIR reflectance intensities across the entire spectral curve for the experiment with three treatments. Using this approach, samples from the experiment with three treatments with reflectance values at 1772 nm less than 150 or greater than 350 were excluded. In total, 8.0% of spectra across all treatment groups (*N* = 389) were removed from the experiment containing three treatments. In the experiment containing two treatments, spectra that resembled the backside of the mirror were manually removed prior to performing outlier detection and trimming. Including those spectra, 14.8% of spectra across all treatment groups (*N* = 210) were removed from the experiment containing two treatments. Note, spectra from seedlings that failed to develop disease symptoms were excluded from pre-processing and subsequent analyses.

Next, spectra were second derivative transformed (package: “mdatools”; width of filter window = 15, porder = 2, and dorder = 2) [33], and data were randomly split into training (70% of data) and testing (30% of data) sets, while maintaining the proportion of each treatment group in each data set (package: “caret”) [34] (Table 1, Table S1). Since NIR spectra are known to contain multicollinear variables, which may result in model overfitting, variable reduction was performed using two methods. First, variable selection using random forests (package: “VSURF”) [35] was used to identify individual spectral bands that are associated with the response (i.e., treatments). With VSURF, two sets of variables are identified: interpretation step and prediction step. Both sets of variables are related to the response, but interpretation step variables may have more redundancy than prediction step variables [36]. Second, spectral resampling, i.e., signal binning, was used to reduce the number of total bands included in the analysis from 55 to 11 (package: “prospectr”, binsize = 5) [30, 37]. A bin size of five was selected to reduce multicollinearity without adversely impacting model performance (i.e., classification accuracies) by decreasing the number of bands too severely.

Supervised classification models were developed using support vector machine (SVM) with scaling (package: “e1071”) [38] and random forest (package: “VSURF”) [35]. Optimal model parameters for SVM were determined using 10-fold cross-validation (Table 2, Table S2), while default parameters were used for the random forest models. Model performance was assessed based on total accuracy from training and testing sets (package: “MLmetrics”) [39], 10-fold cross-validated accuracy on the training set (for SVM only), and for models containing only two treatment groups, receiver operating characteristic (ROC) curves (for SVM only) (package: “ROCR”) [40].

Finally, sparse partial least squares discriminant analysis (sPLS-DA) (package: “mixOmics”) [41] was run to confirm the identities of important spectral bands across analyses and experiments. sPLS-DA not only develops a model for predicting the group of new samples but also identifies bands that are most predictive or important for discriminating between groups. Five-fold cross-validation (repeated 50 times) of the training set was used to identify the optimal number of components (four and three for the experiment with three and two treatments, respectively) and variables for each component that discriminated between control and inoculated groups (mock-inoculated samples were excluded since the treatment group was only present in the second experiment). The accuracy of sPLS-DA model predictions was assessed based on the proportion of samples correctly classified in the testing set and based on the balanced error rate (BER) of prediction of the testing set.

All analyses were based on the spectral range from 1898–2551 nm (55 total bands), which were selected based on spectral absorbance. Spectra with negative absorbance values, likely due to detector signal saturation (a result of external lighting and/or due to the gold-plated mirror used as the background), were excluded from analysis (spectra < 1898nm).

## 3. Results

### 3.1. Disease Phenotyping

**Figure 1:**
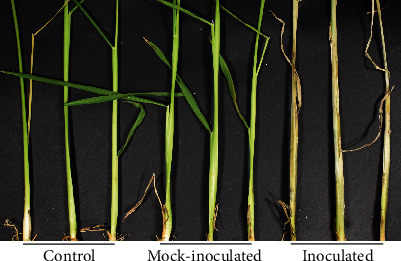
ShB symptomatic rice plants. Representative examples of control, mock-inoculated, and inoculated Lemont rice seedlings at seven days post-inoculation.

All inoculated plants developed symptoms of ShB, i.e., stem lesions, with the exception of three inoculated plants in the experiment containing only two treatments, while mock-inoculated and control plants did not show any symptoms of ShB (Figure 1). The average proportion ± standarderror of inoculated stems covered by lesions (lesion length in cm/stem length in cm) was 0.70 ± 0.01 and 0.90 ± 0.01 at five and seven dpi, respectively (*N* = 76), in the experiment with three treatments, and 0.65 ± 0.02 and 0.80 ± 0.03 at seven and nine dpi, respectively (*N* = 64, with three seedlings that failed to develop disease symptoms removed), in the experiment with two treatments.

### 3.2. NIR Spectra

**Figure 2:**
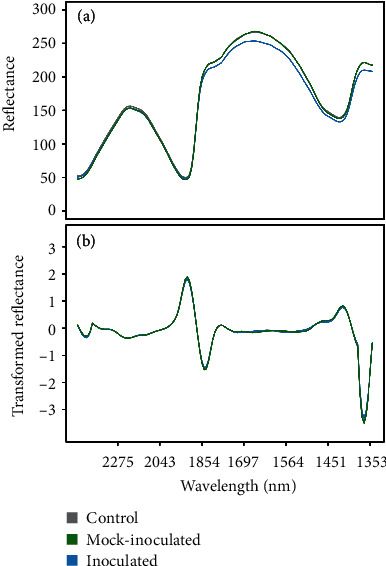
Rice NIR spectra. Average (a) raw and (b) second derivative transformed near-infrared spectra from 2551–1348 nm for control (grey), mock-inoculated (green), and inoculated (blue) Lemont rice seedlings at one-day postinoculation.

Average raw and second derivative transformed spectra for each treatment group can be found in Figure 2 (for the experiment with control, mock-inoculated, and inoculated seedlings) and Figure S1 (for the experiment with control and inoculated seedlings only). Spectra were comparable between the two experiments, although the average intensity of the spectral reflectance was higher in the experiment containing control, mock-inoculated, and inoculated seedlings. There was no observable difference in the overall shape of NIR spectra between treatment groups, although differences were observed in the average intensity of NIR reflectance in some spectral regions (e.g., ~1854–1300 nm and ~ 1854–1564 nm, for the experiment with three and two treatments, respectively) (Figure 2, Figure S1). However, this region was characterized by negative absorbance values and subsequently was excluded from the machine learning analysis.

### 3.3. Variable Selection and Classification Models

The accuracy of classifications from SVM varied depending on the model (i.e., all groups, mock-inoculated versus inoculated, or control versus inoculated) and whether or not a variable reduction method, e.g., VSURF (Table 3, Table S3) or spectral resampling, was used (Table 4, Table S4). The trimmed spectral range from 1898–2551 nm contained 55 total bands. With spectral resampling, this was reduced to 11 bands, and with VSURF, the number of bands varied from as few as four (control versus inoculated comparison in the experiment with three treatments) to 33 (interpretation step variables, all groups comparison) (Table 3, Table S3). Across all analyses, only one VSURF-selected band (2442 nm) was shared at the prediction step.

**Figure 3:**
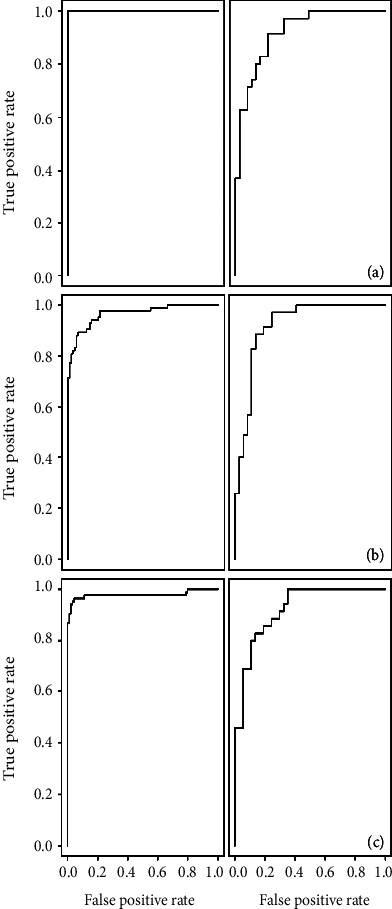
ROC curves. Receiver operating characteristic (ROC) curves for training (left) and testing (right) sets for the SVM classification model for mock-inoculated and inoculated seedlings based on (a) second derivative transformed spectra, (b) spectral bands selected by VSURF, and (c) resampled spectra for the experiment containing control, mock-inoculated, and inoculated seedlings.

**Figure 4:**
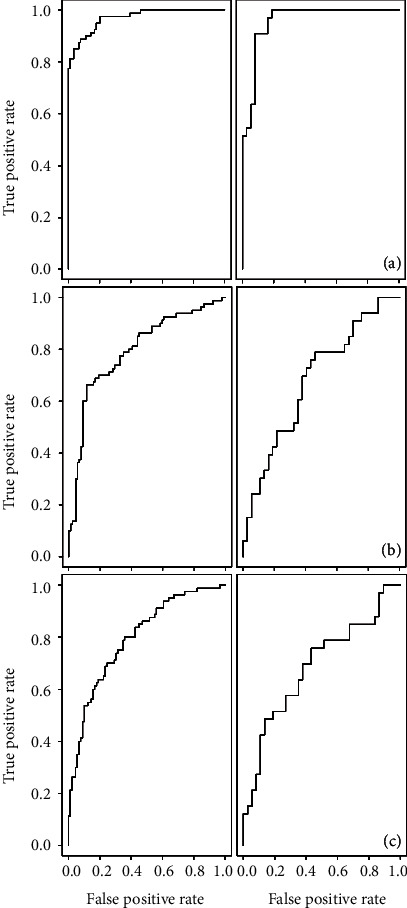
ROC curves. Receiver operating characteristic (ROC) curves for training (left) and testing (right) sets for the SVM classification model for control and inoculated seedlings based on (a) second derivative transformed spectra, (b) spectral bands selected by VSURF, and (c) resampled spectra for the experiment containing control, mock-inoculated, and inoculated seedlings.

When comparing mock-inoculated and inoculated plants, both SVM models based on resampled variables and VSURF prediction step selected variables yielded models with greater than 80% total accuracy for the 10-fold cross-validated training set and the testing set. In both instances, models were better at accurately classifying spectra from inoculated plants. Similar levels of total accuracy (from 60.4–86.8% for the testing data set) for the SVM models were observed for the experiment containing only two treatments (control and inoculated seedlings), with the exception of the SVM model built based on the VSURF-selected bands from the experiment containing three treatments (exp. 2). In that instance, testing and cross-validated training total accuracies were 60.4% and 57.9%, respectively. Classification models from random forests were not as accurate as SVM-based classification models (Table 5, Table S5), although the testing accuracy was only slightly reduced (79.2%–80.6%, *N* = 72) for the mock-inoculated versus inoculated comparison (Table 5). Receiver operating characteristic (ROC) curves were also generated to assess SVM model classification performance for those models only comparing two treatments—mock-inoculated versus inoculated (Figure 3) and control versus inoculated (Figure 4, Figure S2).

Finally, spectral features and regions identified as being important by VSURF were confirmed using sPLS-DA. The ability to identify presymptomatic infected rice plants was also confirmed using sPLS-DA. Several bands identified as being important for distinguishing between groups were shared between VSRUF and sPLS-DA analyses (e.g., 2153, 2200, and 2288 nm) (Table 3, Table S3, Table S6). In addition, 64% of samples in the testing set were correctly classified using sPLS-DA in both experiments (Table 6).

## 4. Discussion

Presymptomatic disease detection based on NIR spectral profiles was achieved for rice plants artificially inoculated with the fungus *R. solani* under growth chamber conditions. NIR spectra were collected one day following inoculation, three days before symptoms first developed, and in tissues away from the site of inoculation. This suggests that systemic changes are occurring inside the plant following pathogen infection, and that NIR spectroscopy combined with machine learning is sensitive enough to detect those changes. As a result, this approach shows great promise as a tool for early detection of this and likely other economically important plant diseases.

While there were no obvious differences in the average spectral profiles of control, mock-inoculated, or inoculated plants from 1898–2551 nm (the NIR region of focus for this study), the SVM model based on VSURF-selected variables correctly identified 94.6% of inoculated plants in the testing (validation) set. For models built using only inoculated and control plants, the overall testing accuracy ranges were comparable across the two experiments; 64.3–88.6% and 60.4–86.8% for the experiments containing three and two treatments, respectively. SVM models built using second derivative transformed NIR spectra from 1898–2551 nm had higher total testing accuracies compared to SVM models built using bands selected by VSURF or from resampled data, except for the mock-inoculated versus inoculated comparison. The ability to classify plants based on inoculation status using NIR spectra was also evaluated using random forest (VSURF). Random forest models could be used to classify rice based on NIR spectra, although these models were not as accurate as SVM models, perhaps due in part to the fact that parameters for SVM models were optimized, while default parameters were used for VSURF models. Furthermore, the levels of accuracy in our study are in-line with other studies describing spectroscopy as a tool for early disease detection. For example, Rumpf et al. [18] and Arens et al. [42] used hyperspectral data to classify infected sugar beets prior to the development of visible symptoms of *Cercospora* leaf spot. Accuracy levels in these studies ranged from 65–80% [18] to 98.5–99.9% [42].

To examine the transferability of results between experiments, the VSURF-selected variables from the experiment containing all three treatments were used to classify plants from the experiment containing only two treatments. Since spectra were collected in slightly different manners, data from the two experiments were not combined. The SVM model based on these variables could correctly classify control and inoculated plants in the testing set only 60.4% of the time. This lower accuracy could be explained by differences in the manner in which we collected spectral data across the two experiments, but also could be attributed to variation in environmental conditions across the two experiments [43]. This includes variation in the ambient environmental conditions under which spectra were collected and also the environmental conditions under which plants were grown and inoculated. For instance, even though plants were grown under the same growth chamber conditions, we observed differences in the rate of disease development between experiments. Since the development of ShB is influenced by humidity and temperature [21, 44, 45], environmental conditions likely varied slightly between the two experiments. It is also possible that environmental variation influenced the physiology of the plant and thus their spectral profiles, and subsequently the results of our machine learning analysis. While this may explain in part the different selection of variables by VSURF, it did not impact our ability to build and validate accurate models for classifying infected plants prior to symptom development.

To our knowledge, this is the first study to use NIR spectroscopy to classify ShB-infected rice prior to the development of symptoms, although a study by Wang et al. [11] used NIR spectroscopy to detect differences in the severity of ShB infections. They found that rice reflectivity changed as ShB damage increased, and postulated that changes in reflectance, particularly between 1900 and 2000 nm, could be associated with water loss [11]. Even though we did not observe any pronounced differences between the average NIR spectral profiles of inoculated and control plants between 1900 and 2000 nm, VSURF and sPLS-DA identified several bands in that region (e.g., 1916, 1944, 1953, and 1982 nm), which are also close in position to 1940 nm, a band known to be associated with water [46]. Therefore, our ability to identify presymptomatic plants may be linked to early changes in plant moisture content in infected compared to healthy rice plants. Early changes in the physiochemistry of rice plants following infection with *R. solani* may also be linked to changes in starch, cellulose, protein, and nitrogen content. Bands at 2097, 2200, and 2288 nm were identified as being important for classifying samples across analyses (VSURF and sPLS-DA) and experiments and are close to bands associated with those groups as reported by Curran (e.g., 2100, 2180, 2240, and 2300 nm) [46]. Furthermore, changes in the expression of cell-wall degrading genes and cellulose-degrading enzymes in *R. solani* are known to occur in the early stages of plant infection [47]. Since we recorded spectra at one-day following inoculation, it is possible that we detected changes in spectral reflectance associated with the *R. solani* pathogenesis process.

In conclusion, NIR spectroscopy, combined with machine learning, shows great potential as a tool for early detection of the presymptomatic state of infected plants. Specifically, we demonstrated that rice infected with *R. solani* can be detected as early as one day following inoculation, in tissues away from the site of inoculation, and prior to symptom development. Early detection of ShB may allow for more rapid and targeted disease management, saving both time and money. In addition, the tool has the capacity to be used in a high-throughput manner (current collection times are only two seconds per leaf) and could be used in combination with unmanned aerial vehicles (UAVs or drones). Since the current study examined only growth chamber grown and artificially inoculated rice, future studies should focus on evaluating the use of NIR spectroscopy combined with machine learning as a tool for ShB detection in field settings and for differentiating between multiple biotic and abiotic stressors.

## Figures and Tables

**Table 1:** Sample sizes. Data were randomly split into training (70% of data) and testing (30% of data) sets for model development and validation for the experiment containing control, mock-inoculated, and inoculated seedlings.

Comparison	Data set	Total *N*		
		Control	Mock-inoculated	Inoculated
All groups	Training	80	84	89
	Testing	33	35	37
Mock vs. inoculated	Training	—	84	89
	Testing	—	35	37
Control vs. inoculated	Training	80	—	89
	Testing	33	—	37

**Table 2:** SVM model parameters. Support vector machine (SVM) optimal parameters for the experiment containing three treatments (control, mock-inoculated, and inoculated seedlings).

Comparison	Model	Kernel	Cost	Gamma
All groups	Second derivative	Linear	100	—
	VSURF	Radial	100	0.05
	Resampled	Radial	100	0.05
Mock vs. inoculated	Second derivative	Radial	100	0.05
	VSURF	Radial	100	0.05
	Resampled	Radial	100	0.05
Control vs. inoculated	Second derivative	Linear	100	—
	VSURF	Linear	0.1	—
	Resampled	Linear	0.1	—

**Table 3:** VSURF-selected bands. Variable selection using random forests- (VSURF-) selected bands at prediction and interpretation steps for the experiment containing three treatments (control, mock-inoculated, and inoculated seedlings). Prediction step variables used for support vector machine (SVM) classification models.

Comparison	Selected bands (nm)	
	Prediction step	Interpretation step
All groups	2442, 2054, 2427, 2153, 2356, 2165, 2225, 1982	2442, 2054, 2427, 2153, 2043, 2356, 2165, 2370, 2328, 2033, 2384, 2141, 2176, 2342, 2315, 2119, 2212, 2412, 2275, 2130, 2188, 2225, 2200, 2398, 2302, 2086, 2064, 2075, 2288, 2108, 1992, 2022, 1982
Mock vs. inoculated	2130, 2442, 2275, 2141, 2153, 2328, 2097, 2427, 2200	2130, 2442, 2275, 2141, 2153, 2176, 2328, 2165, 2108, 2288, 2119, 2097, 2427, 2200
Control vs. inoculated	2119, 2370, 2033, 2442	2119, 2370, 2033, 2442

**Table 4:** SVM classification performance. Support vector machine (SVM) classification performance for the experiment containing three treatments (control, mock-inoculated, and inoculated seedlings).

Comparison	Model	Data set	Accuracy	10-fold CV accuracy	Proportion correctly classified		
					Control	Mock-inoculated	Inoculated
All groups	Second derivative	Training	0.822	0.708	0.825	0.786	0.854
		Testing	0.733	—	0.788	0.600	0.811
	VSURF	Training	0.830	0.664	0.775	0.810	0.899
		Testing	0.714	—	0.576	0.600	0.946
	Resampled	Training	0.866	0.644	0.813	0.905	0.876
		Testing	0.657	—	0.606	0.600	0.757
Mock vs. inoculated	Second derivative	Training	1.000	0.757	—	1.000	1.000
		Testing	0.806	—	—	0.829	0.784
	VSURF	Training	0.890	0.832	—	0.810	0.966
		Testing	0.861	—	—	0.829	0.892
	Resampled	Training	0.936	0.809	—	0.881	0.989
		Testing	0.847	—	—	0.800	0.892
Control vs. inoculated	Second derivative	Training	0.911	0.811	0.850	—	0.966
		Testing	0.886	—	0.848	—	0.919
	VSURF	Training	0.763	0.746	0.688	—	0.831
		Testing	0.643	—	0.485	—	0.784
	Resampled	Training	0.710	0.704	0.650	—	0.764
		Testing	0.643	—	0.545	—	0.730

**Table 5:** VSURF classification performance. Variable selection using random forests (VSURF) classification performance based on bands selected at prediction and interpretation steps (Table 3) for the experiment containing three treatments (control, mock-inoculated, and inoculated seedlings).

Comparison	Model	Data set	Accuracy	Proportion correctly classified		
				Control	Mock-inoculated	Inoculated
All groups	Prediction	Training	1.000	1.000	1.000	1.000
		Testing	0.562	0.515	0.457	0.703
	Interpretation	Training	1.000	1.000	1.000	1.000
		Testing	0.600	0.485	0.457	0.838
Mock vs. inoculated	Prediction	Training	1.000	—	1.000	1.000
		Testing	0.792	—	0.743	0.838
	Interpretation	Training	1.000	—	1.000	1.000
		Testing	0.806	—	0.743	0.865
Control vs. inoculated	Prediction	Training	1.000	1.000	—	1.000
		Testing	0.657	0.485	—	0.811
	Interpretation	Training	1.000	1.000	—	1.000
		Testing	0.657	0.485	—	0.811

**Table 6:** Prediction performance of sPLS-DA for control vs. inoculated samples. Sparse partial least squares discriminating analysis (sPLS-DA) prediction performance of the testing set. Bands (Table S6) selected during model calibration using the training set for experiment one (Exp. 1; experiment with two treatments) and experiment two (Exp. 2; experiment with three treatments).

Experiment	No. components	BER	Proportion correctly classified		
			Control	Inoculated	Total
Exp. 1 (2 treatments)	3	0.359	0.643	0.640	0.642
Exp. 2 (3 treatments)	4	0.362	0.545	0.730	0.643

## Data Availability

Data is available upon request to the corresponding author.

## Supplementary Materials

Supplementary Materials Figure S1: NIR spectra. Average (a) raw and (b) second derivative transformed near-infrared spectra from 2551–1348 nm for control (grey) and inoculated (blue) Lemont rice seedlings at one day post-inoculation from the experiment containing only control and inoculated seedlings. Figure S2: ROC curves. Receiver operating characteristic (ROC) curves for (left) training and (right) testing sets for the SVM classification models for control and inoculated seedlings based on (a) second derivative transformed spectra, (b) variables selected by VSURF (exp. 1), (c) variables selected by VSURF (exp. 2), and (d) resampled spectra for the experiment containing only control and inoculated seedlings (exp. 1). Table S1: Sample sizes. Data were randomly split into training (70% of data) and testing (30% of data) sets for model development and validation for the experiment containing only control and inoculated seedlings. Table S2: Optimal SVM parameters. Support vector machine (SVM) optimal parameters for the experiment containing only control and inoculated seedlings. Table S3: VSURF-selected bands. Variable selection using random forests- (VSURF-) selected bands at prediction and interpretation steps. Prediction step variables used for support vector machine (SVM) classification models for the experiment containing only control and inoculated seedlings. Table S4: SVM classification performance. Support vector machine (SVM) classification performance for the experiment containing only control and inoculated seedlings. Note, classification performance is indicated for models using VSURF prediction step variables from the experiment only comparing control and inoculated seedlings (exp. 1) and variables selected from the experiment which also contained mock-inoculated seedlings (exp. 2; Table 3, “Control vs. Inoculated”). Table S5: VSURF classification performance. Variable selection using random forests (VSURF) classification performance based on bands selected at prediction and interpretation steps (Table S3) for the experiment containing only control and inoculated seedlings. Table S6: sPLS-DA-selected bands. Bands selected for each component of the sparse partial least squares discriminant analysis (sPLS-DA) model for the experiment with two treatments (Exp. 1) and the experiment with three treatments (Exp. 2) for the control versus inoculated treatments comparison.
