# Supplementary material for: Machine Learning-Based Presymptomatic Detection of Rice Sheath Blight Using Spectral Profiles
Source: Plant Phenomics. 2020 Oct 12;2020:8954085. doi: 10.34133/2020/8954085 (PMC7706329; doi:10.34133/2020/8954085)
Supplement: Supplementary Materials — Figure S1: NIR spectra. Average (a) raw and (b) second derivative transformed near-infrared spectra from 2551–1348 nm for control (grey) and inoculated (blue) Lemont rice seedlings at one day post-inoculation from the experiment containing only control and inoculated seedlings. Figure S2: ROC curves. Receiver operating characteristic (ROC) curves for (left) training and (right) testing sets for the SVM classification models for control and inoculated seedlings based on (a) second derivative transformed spectra, (b) variables selected by VSURF (exp. 1), (c) variables selected by VSURF (exp. 2), and (d) resampled spectra for the experiment containing only control and inoculated seedlings (exp. 1). Table S1: Sample sizes. Data were randomly split into training (70% of data) and testing (30% of data) sets for model development and validation for the experiment containing only control and inoculated seedlings. Table S2: Optimal SVM parameters. Support vector machine (SVM) optimal parameters for the experiment containing only control and inoculated seedlings. Table S3: VSURF-selected bands. Variable selection using random forests- (VSURF-) selected bands at prediction and interpretation steps. Prediction step variables used for support vector machine (SVM) classification models for the experiment containing only control and inoculated seedlings. Table S4: SVM classification performance. Support vector machine (SVM) classification performance for the experiment containing only control and inoculated seedlings. Note, classification performance is indicated for models using VSURF prediction step variables from the experiment only comparing control and inoculated seedlings (exp. 1) and variables selected from the experiment which also contained mock-inoculated seedlings (exp. 2; Table 3, “Control vs. Inoculated”). Table S5: VSURF classification performance. Variable selection using random forests (VSURF) classification performance based on bands selected at prediction and int [file 8954085.f1.docx]

**Supplementary Materials**

Machine learning-based presymptomatic detection of rice sheath blight using spectral profiles

Anna O. Conrad^1^*, Wei Li^1^, Da-Young Lee^1^, Guo-Liang Wang^1^, Luis Rodriguez-Saona^2^, and Pierluigi Bonello^1^

^1^Department of Plant Pathology, The Ohio State University, Columbus, Ohio, USA

^2^Department of Food Science and Technology, The Ohio State University, Columbus, Ohio, USA

*Corresponding author. Email: conrad.245@osu.edu

**Supplementary Figures and Tables**


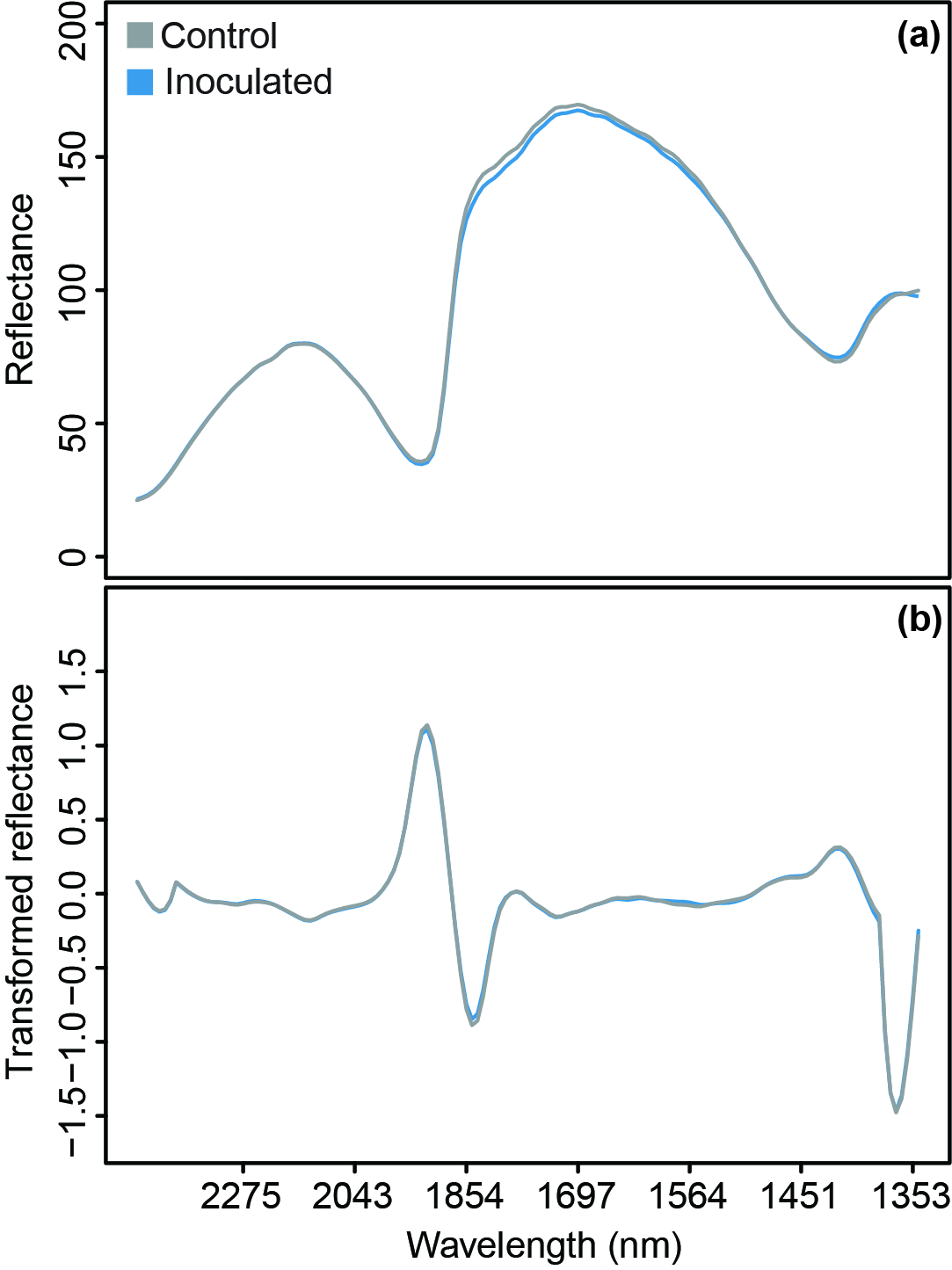


**Figure S1. NIR spectra.** Average (a) raw and (b) second derivative transformed near-infrared spectra from 2551 – 1348 nm for control (grey) and inoculated (blue) Lemont rice seedlings at one day post-inoculation from the experiment containing only control and inoculated seedlings.


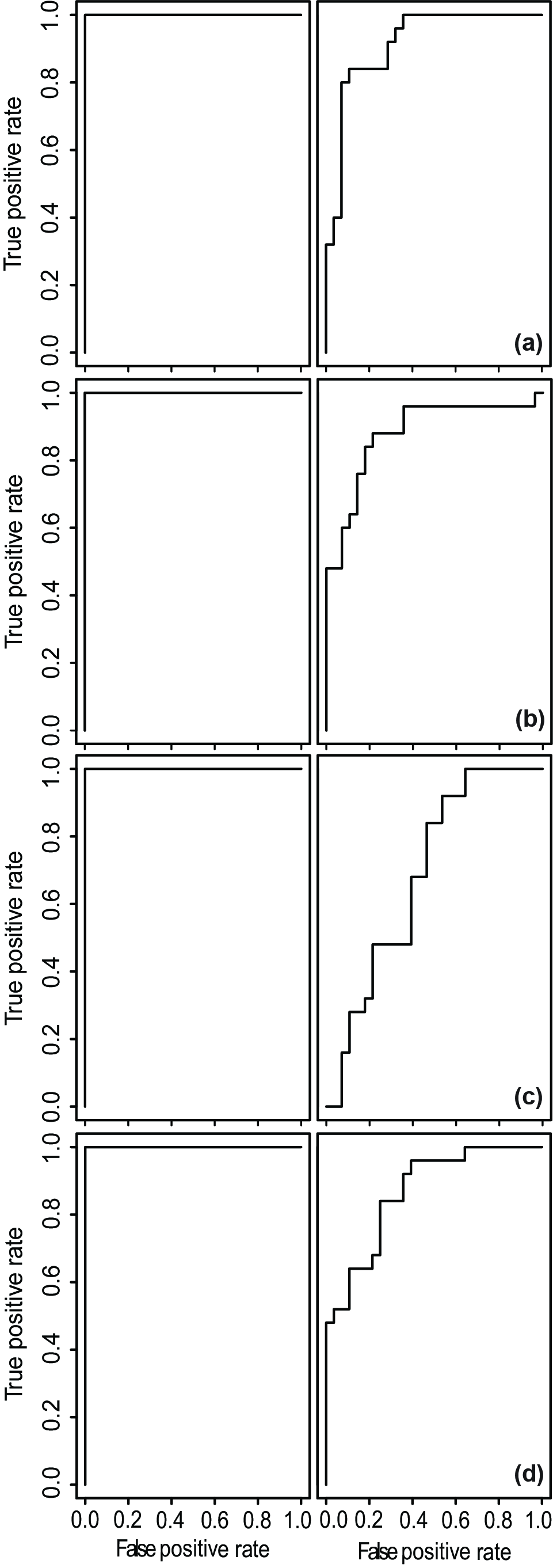


**Figure S2. ROC curves.** Receiver operating characteristic (ROC) curves for (left) training and (right) testing sets for the SVM classification models for control and inoculated seedlings based on (a) second derivative transformed spectra, (b) variables selected by VSURF (exp. 1), (c) variables selected by VSURF (exp. 2), and (d) resampled spectra for the experiment containing only control and inoculated seedlings (exp. 1).

**Table S1. Sample sizes.** Data were randomly split into training (70% of data) and testing (30% of data) sets for model development and validation for the experiment containing only control and inoculated seedlings.

| Comparison | Data set | Total N | |
| --- | --- | --- | --- |
|  |  | Control | Inoculated |
| Control vs. Inoculated | Training | 66 | 60 |
|  | Testing | 28 | 25 |

**Table S2. Optimal SVM parameters.** Support vector machine (SVM) optimal parameters for the experiment containing only control and inoculated seedlings.

| Comparison | Model | Kernel | Cost | Gamma |
| --- | --- | --- | --- | --- |
| Control vs. Inoculated | Second derivative | radial | 10 | 0.05 |
|  | VSURF (exp. 1) | radial | 100 | 0.5 |
|  | VSURF (exp. 2) | radial | 100 | 5 |
|  | Resampled | radial | 1 | 0.5 |

**Table S3. VSURF-selected bands.** Variable selection using random forests (VSURF)-selected bands at prediction and interpretation steps. Prediction step variables used for support vector machine (SVM) classification models for the experiment containing only control and inoculated seedlings.

| Comparison | Selected bands (nm) | |
| --- | --- | --- |
|  | Prediction step | Interpretation step |
| Control vs. Inoculated | 2288, 1916, 1944, 2097, 2442, 1953, 2250 | 2288, 1916, 1925, 1935, 1944, 2097, 2086, 2551, 2075, 2442, 2064, 2302, 1953, 2250, 2427, 2200, 2153 |
|  |  |  |
|  |  |  |
|  |  |  |

**Table S4. SVM classification performance.** Support vector machine (SVM) classification performance for the experiment containing only control and inoculated seedlings. Note, classification performance is indicated for models using VSURF prediction step variables from the experiment only comparing control and inoculated seedlings (exp. 1) and variables selected from the experiment which also contained mock-inoculated seedlings (exp. 2; **Table 3**, “Control vs. Inoculated”).

| Comparison | Model | Data set | Accuracy | 10-fold CV accuracy | Proportion correctly classified | |
| --- | --- | --- | --- | --- | --- | --- |
|  |  |  |  |  | Control | Inoculated |
| Control vs. Inoculated | Second derivative | Training | 1.000 | 0.698 | 1.000 | 1.000 |
|  |  | Testing | 0.868 | -- | 0.929 | 0.800 |
|  | VSURF (exp. 1) | Training | 1.000 | 0.722 | 1.000 | 1.000 |
|  |  | Testing | 0.774 | -- | 0.857 | 0.680 |
|  | VSURF (exp. 2) | Training | 1.000 | 0.579 | 1.000 | 1.000 |
|  |  | Testing | 0.604 | -- | 0.714 | 0.480 |
|  | Resampled | Training | 0.992 | 0.730 | 1.000 | 0.983 |
|  |  | Testing | 0.736 | -- | 0.821 | 0.640 |

**Table S5. VSURF classification performance.** Variable selection using random forests (VSURF) classification performance based on bands selected at prediction and interpretation steps (**Table S3**) for the experiment containing only control and inoculated seedlings.

| Comparison | Model | Data set | Accuracy | Proportion correctly classified | |
| --- | --- | --- | --- | --- | --- |
|  |  |  |  | Control | Inoculated |
| Control vs. Inoculated | Prediction | Training | 1.000 | 1.000 | 1.000 |
|  |  | Testing | 0.604 | 0.679 | 0.520 |
|  | Interpretation | Training | 1.000 | 1.000 | 1.000 |
|  |  | Testing | 0.698 | 0.679 | 0.720 |

**Table S6. sPLS-DA-selected bands.** Bands selected for each component of the sparse partial least squares discriminant analysis (sPLS-DA) model for the experiment with two treatments (Exp. 1) and the experiment with three treatments (Exp. 2) for the control versus inoculated treatments comparison.

| Experiment | Component | Bands* |  |
| --- | --- | --- | --- |
| Exp. 1 | 1 | 2302, 2315, 2288, 1898, 1907, 2328, 1916, 1925, 1935, 1944, 1953, 2342, 2275, 2012, 2022 |  |
|  | 2 | 2002, 2503, 2012, 2487, 2519, 2472, 1992, 2457, 2022, 2535, 2153, 2033, 2398, 2412, 2165 |  |
|  | 3 | 2176, 2165, 2188, 2153, 2200, 2212 |  |
| Exp. 2 | 1 | 2356, 2342, 2108, 2119, 2370, 2328, 2130, 2097, 2141, 2022, 2012, 2442, 2384, 2033, 2315, 2002, 2153, 2086, 1992, 2043, 2165, 1982, 2302, 1973, 2398 |  |
|  | 2 | 1898, 2250, 2237, 2225, 2263, 2212, 2551, 2200, 1907, 2275, 1953, 2043, 1963, 2188, 1916, 1944, 2033, 1925, 1935, 2054, 1973, 2176, 2535, 2288, 2064, 2022, 2398, 2412, 2519, 2165, 2503, 2384, 1982, 2487, 2472 |  |
|  | 3 | 2033, 2022, 2043, 2012, 2054 |  |
|  | 4 | 2165, 2153, 2176, 2141, 2398, 2384, 2075, 2188, 2130, 2370, 2086, 2064, 2412, 2275, 2288 |  |
| *Selected bands for each component. Bands listed in order from greatest to least absolute weight coefficient. | | |  |
|  |  |  |  |
